# PI3K/AKT signaling pathway: new strategies for treating atherosclerosis with plant-derived compounds

**DOI:** 10.3389/fphar.2026.1722493

**Published:** 2026-03-19

**Authors:** Lin Zhu, Qiuhan Chen, Boyu Wang, Jiamei Fu, Zhiping Liu, Yingying Cui, Ruiting Zhang, Fengwei Liu, Siying Niu, Yabin Zhou

**Affiliations:** 1 First Clinical Medical School, Heilongjiang University of Chinese Medicine, Harbin, Heilongjiang, China; 2 Department of Cardiology, First Hospital of Heilongjiang University of Chinese Medicine, Harbin, Heilongjiang, China; 3 Department of Geriatrics, First Hospital of Heilongjiang University of Chinese Medicine, Harbin, Heilongjiang, China; 4 Fever Clinic, First Hospital of Heilongjiang University of Chinese Medicine, Harbin, Heilongjiang, China

**Keywords:** atherosclerosis, inflammation, PI3K/AKT signaling pathway, plant-derived compounds, review

## Abstract

Atherosclerosis (AS) is a chronic vascular inflammatory disease driven by lipid deposition, whose clinical management remains constrained by the limitations of existing pharmacological interventions. This review systematically elucidates the molecular mechanisms by which plant-derived compounds modulate AS through targeted regulation of the phosphoinositide-3-kinase/protein kinase B (PI3K/AKT) signaling pathway. Studies indicate that plant-derived compounds—such as terpenoids (e.g., artemisinin and tanshinone IIA) and alkaloids (e.g., berberine)—effectively attenuate the progression of AS via bidirectional modulation of the PI3K/AKT pathway. In early stages, suppression of this pathway downregulates downstream mechanistic target of rapamycin (mTOR) and nuclear factor-kappa B (NF-κB) protein expression, thereby mitigating inflammatory responses and lipid accumulation to inhibit plaque formation. Conversely, during advanced disease phases, moderate activation of the pathway upregulates key effectors, including autophagy-related protein (Beclin-1), glutathione (GSH), and glutathione peroxidase 4 (GPX4), promoting ferroptosis and autophagy in abnormal cells and thereby enhancing the stability of established plaques. It is noteworthy that the low bioavailability of plant-derived compounds and the stage-specific nature of pathway modulation remain critical challenges for clinical translation. In this review, we deepen the mechanistic understanding of plant-based interventions against AS and provide a theoretical foundation and innovative perspectives for the development of future botanically derived AS therapeutics.

## 1 Introduction

Atherosclerosis (AS) is a chronic vascular inflammatory disease primarily driven by the accumulation of lipoproteins, predominantly affecting medium- and large-sized arteries (Pan et al., 2024; Tabares-Guevara et al., 2021). The progression of AS is defined by the accumulation of cholesterol-rich plaques within the arterial endothelium, thereby inducing narrowing and potential occlusion of the vascular lumen (Ajoolabady et al., 2024; Fan and Watanabe, 2022). Epidemiological studies indicate that ischemic heart disease resulting from AS is the leading cause of global disability-adjusted life years (DALYs), with an age-standardized rate of 2,275.9 per 100,000 persons, and mortality continues to increase, particularly in Eastern European and Asian populations (Herrington et al., 2016; Mensah et al., 2023). Major risk factors for AS include hypertension, hypercholesterolemia, and diabetes (Xing and Lin, 2025). The pathogenesis of AS involves prolonged stimulation by abnormal lipid metabolism, which induces endothelial cell injury and activates the immune system, leading to foam cell formation (Liu et al., 2020). These foam cells secrete a wide range of inflammatory mediators. Concurrently, vascular smooth muscle cells undergo phenotypic switching, migrating from the medial layer to the intima, where they proliferate, exacerbating the deterioration of the vascular microenvironment and thereby driving the development of AS (Meng et al., 2022; Tang et al., 2022; Zhang L. et al., 2024). This pathological process involves multiple signaling pathways, among which the phosphoinositide-3-kinase/protein kinase B (PI3K/AKT) pathway plays a critical role due to its central function in regulating immune responses and lipid metabolism, making it particularly significant in the progression of AS (Cui et al., 2023; Zheng et al., 2020; Zhou M. et al., 2019).

The PI3K/AKT signaling pathway represents a highly conserved signal transduction cascade widely present in mammalian cells (Aytenfisu et al., 2022; Guerau-de-Arellano et al., 2022; Karar and Maity, 2011). As an intracellular phosphatidylinositol kinase, PI3K modulates the activity of AKT and glycogen synthase kinase 3 (GSK3), thereby influencing autophagy and platelet function (Liu Y. et al., 2023; Wang et al., 2022). AKT, a member of the serine–threonine kinase family, plays a pivotal role in regulating metabolism, cell differentiation, and proliferation (Hassan et al., 2024; Miao et al., 2022; Yudushkin, 2019). Accumulating evidence in recent years has shown that modulation of the PI3K/AKT signaling pathway enhances the activity of key mediators responsible for vascular endothelial homeostasis and promotes macrophage polarization, suggesting its potential to attenuate the initiation and progression of AS (Liberale et al., 2023; Manning and Toker, 2017; Wang et al., 2023; Wang Y. et al., 2025).

**FIGURE 1:**
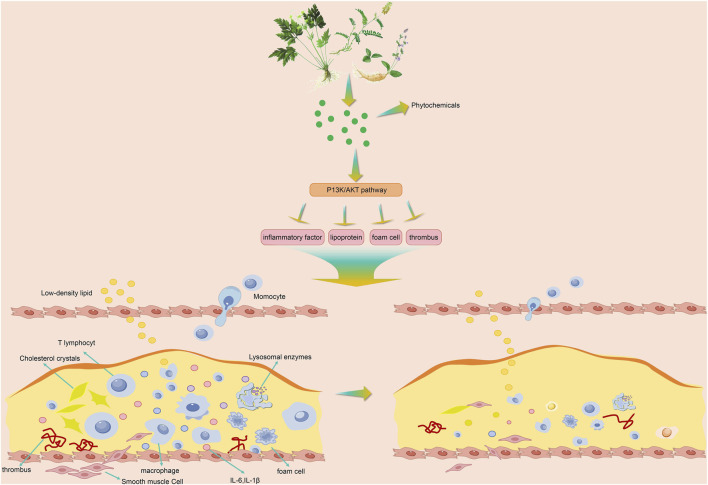
Effects of plant-derived compounds on AS.

Current mainstream therapeutic strategies for AS predominantly involve oral administration of statins and nitrate drugs, along with interventional procedures such as percutaneous coronary intervention (PCI) (Erbel et al., 2014; Sarraju and Nissen, 2024). Although these approaches significantly control the progression of AS, they are nonetheless accompanied by certain side effects (Chen et al., 2025; Ruscica et al., 2023). Consequently, there is a pressing need to explore alternative therapies with improved safety profiles and reduced toxicity for AS treatment. In recent years, natural plant-derived compounds have gained increasing attention in global clinical applications due to their accessibility and favorable toxicity characteristics (Wang A. et al., 2024; Wang B. et al., 2025; Wang L. et al., 2024; Wang Z. C. et al., 2021). Plant-derived compounds, which are chemically diverse substances extracted from terrestrial or marine plants, primarily include terpenoids, flavonoids, and alkaloids. Accumulating evidence indicates that these plant-derived compounds inhibit the development of AS through pleiotropic mechanisms—such as antioxidant, anti-inflammatory, and lipid-lowering effects—mediated via multiple signaling pathways. For instance, Arab et al. (2022) demonstrated that specific plant-derived compounds upregulate paraoxonase 1 (PON1) activity, thereby preventing the oxidation of low-density lipoprotein (LDL) and inhibiting foam cell formation at early stages. Similarly, Centner et al. (2023) confirmed that modulation of gut microbiota by plant-derived compounds leads to reduced circulating triglycerides (TGs) and total cholesterol (TC) while increasing high-density lipoprotein (HDL) levels. Further mechanistic studies have demonstrated that anthocyanins activate nuclear factor erythroid 2-related factor 2 (Nrf2) and downstream antioxidant genes, thereby exerting anti-inflammatory and antioxidant effects that effectively suppress AS progression (Xin et al., 2024). Additionally, wogonin upregulates peroxisome proliferator-activated receptor alpha (PPARα) expression, which enhances cholesterol efflux and inhibits macrophage foam cell formation, thereby highlighting its considerable potential for AS therapeutic development (Ma et al., 2025). In this review, we focus on the PI3K/AKT signaling pathway as a central axis to summarize the mechanisms and targets of plant-derived compounds in the treatment of AS and provide new therapeutic perspectives for future research on AS (Figure 1).

Plant-derived compounds exert their potential protective effects by modulating the PI3K/AKT signaling pathway, thereby simultaneously intervening in multiple aspects of the AS process, including the inflammatory response, lipid metabolism, foam cell formation, and thrombogenesis, and demonstrating multi-target and coordinated mechanisms of action.

## 2 Research methods

A comprehensive literature search was systematically conducted across six electronic databases—PubMed, MEDLINE, Scopus, Embase, CNKI, and Web of Science—using keywords such as “plant-derived compounds,” “atherosclerosis,” “PI3K,” “AKT,” “endothelial cells,” and “botanical drug,” along with relevant MeSH terms. Studies published between January 2020 and December 2025 were included, with further selection prioritizing those reporting explicit experimental parameters, such as dosage range, minimum active concentration, treatment duration, and model specifications. These selected studies were organized using the reference management software EndNote. Initially, 872 articles were identified through the search strategy. The rigorous screening process effectively screened out studies with potential risks of reporting bias, detection bias, selection bias, or other sources of bias. Ultimately, 83 articles met the eligibility criteria and were incorporated into this review.

## 3 Relationship between the PI3K/AKT signaling pathway and AS

PI3K, a key intracellular enzyme in the PI3K/AKT signaling pathway, is primarily stimulated by G protein-coupled receptors (GPCRs), receptor tyrosine kinases (RTKs), and various growth factors. Upon activation, PI3K facilitates the phosphorylation of AKT through a series of biochemical reactions (Guo et al., 2024; Yu et al., 2023). Phosphorylated AKT subsequently modulates downstream targets such as mTOR and GSH, thereby serving as a pivotal node in controlling cellular metabolism, apoptosis, and macrophage polarization (Linton et al., 2019). These molecular events critically influence essential conditions for AS progression, including the pro-inflammatory intravascular environment and plaque vulnerability (Wang et al., 2022; Zhao et al., 2021). AS represents a distinct form of inflammatory pathology, characterized by persistent low-grade inflammation that occurs from the initial subclinical stages through the advanced complication phases of the disease (Attiq et al., 2024; Gusev and Sarapultsev, 2023; Kong et al., 2022). Suppression of the PI3K/AKT signaling pathway has been reported to downregulate the expression of active factors such as vascular cell adhesion molecule-1 (VCAM-1) and monocyte chemoattractant protein-1 (MCP-1), as well as cluster of differentiation 29 (CD29) on monocytes, thereby alleviating endothelial inflammation (Ding et al., 2024; Wang Q. et al., 2024; Zegeye et al., 2018). Macrophages constitute the most abundant leukocyte subpopulation during AS progression. Among them, M1-polarized macrophages sustain prolonged inflammatory activation and are more prone to generating pro-inflammatory cytokines than M2 macrophages (Soehnlein and Libby, 2021; Wen et al., 2022). Inhibition of the PI3K/AKT pathway has been demonstrated to reduce the production of TNF-α, MCP-1, and interleukin-6 (IL-6), while promoting macrophage polarization toward the M2 phenotype (Liu X. et al., 2024). Oxidative stress generates substantial amounts of reactive oxygen species (ROS), which mediate multiple programmed cell death pathways, including ferroptosis, necroptosis, and pyroptosis, which are instrumental in AS pathogenesis (Batty et al., 2022; Hu et al., 2022; Zheng et al., 2022). One study indicated that modulation of the PI3K/AKT signaling pathway effectively suppresses ROS generation by attenuating endoplasmic reticulum stress, thereby protecting endothelial cells from oxidative injury (Lin et al., 2020).

The elevation of circulating lipoproteins and lipids constitutes a key mechanism in the advancement (Lampsas et al., 2023) of AS (Xiang et al., 2022). Downregulation of PI3K/AKT phosphorylation has been demonstrated to downregulate the expression of sterol regulatory element-binding protein 1 (SREBP-1), thereby reducing plasma TG, TC, and LDL levels (Liu R. et al., 2023; Wang X. et al., 2025). One study revealed that attenuated PI3K/AKT signaling following Zdhhc1 gene knockout led to decreased lipid deposition and cholesterol accumulation, along with reduced serum TG and TC concentrations (Zhou et al., 2025). Appropriate autophagy activation helps protect blood vessels from inflammatory and oxidative damage (Ding et al., 2025; Fang et al., 2021; Lin et al., 2021). Modulation of the PI3K/AKT/mTOR axis has been shown to enhance the expression of autophagy-related protein (Beclin-1) and UNC-51-like kinase 1 (ULK1), thereby promoting autophagic activity (Shu et al., 2024). Furthermore, suppressing the PI3K/AKT/mTOR axis inhibits the abnormal proliferation and migration of vascular smooth muscle cells, thereby helping maintain intracellular homeostasis and contributing to the attenuation of AS progression (Shu et al., 2024). To summarize, therapeutic targeting of the PI3K/AKT signaling pathway can effectively mitigate AS development through its ability to modulate lipid metabolism, suppress oxidative stress and inflammation, enhance autophagy, and induce macrophage polarization.

**FIGURE 2:**
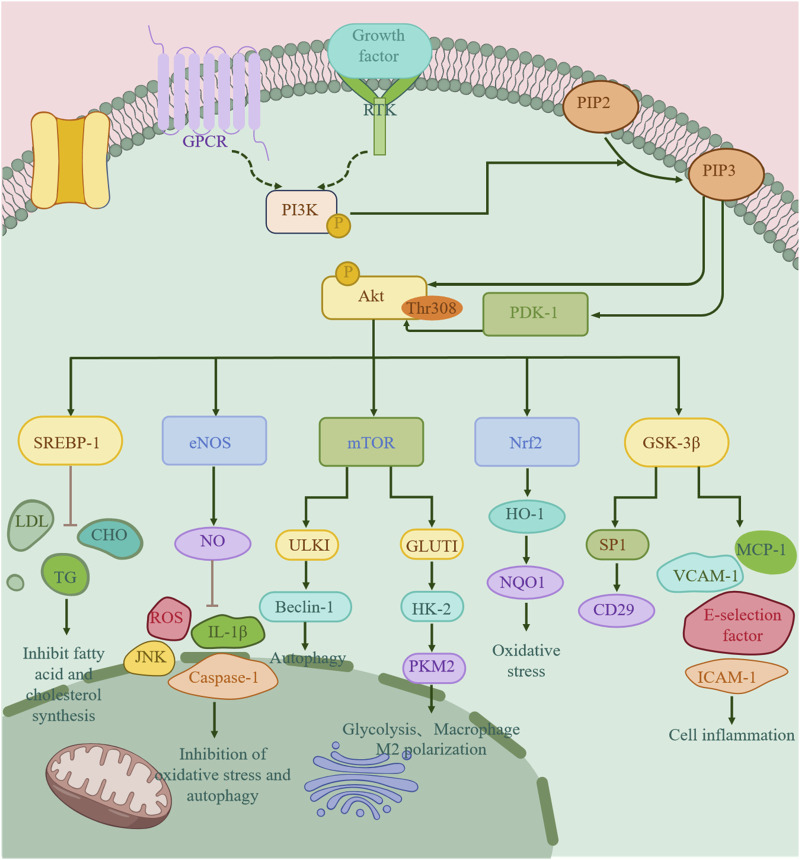
Mechanism of action of the PI3K/AKT signaling pathway in the treatment of AS.

The figure schematically illustrates that upon activation by membrane-bound receptors such as RTKs and GPCRs, PI3K catalyzes the conversion of phosphatidylinositol-4,5-bisphosphate (PIP2) into the second messenger phosphatidylinositol-3,4,5-trisphosphate (PIP3). PIP3 subsequently recruits and activates the key serine/threonine kinase AKT. Serving as a central signaling hub, activated AKT phosphorylates multiple downstream targets to regulate metabolic processes. For instance, it activates Nrf2, initiating the expression of antioxidant genes such as NQO-1 and HO-1, thereby exerting antioxidative effects. Additionally, AKT upregulates eNOS and downstream NO levels, which, in turn, suppresses the production of IL-1β, ROS, and caspase-1, leading to attenuated inflammatory responses. Furthermore, AKT signaling contributes to the inhibition of glycolysis and *de novo* lipid synthesis, collectively influencing the progression of AS. Abbreviations: HO-1, heme oxygenase-1; NQO-1, NAD (P)H quinone dehydrogenase 1; IL-1β, interleukin-1β; NO, nitric oxide; eNOS, endothelial nitric oxide synthase; GSK-3β, glycogen synthase kinase 3β; Caspase-1, cysteine-requiring aspartate protease 1; GLUT1, glucose transporter 1; HK-2, hexokinase 2; JNK, c-Jun N-terminal kinase; PKM2, pyruvate kinase M2; SP1, specificity protein 1 (Figure 2).

## 4 Plant-derived compounds in the treatment of AS

### 4.1 Terpenoids

Terpenoid compounds are ring-shaped or linear structures formed by the rearrangement of the main chain of isopentadiene carbon and are present in almost all plant species (Bergman et al., 2019; Câmara et al., 2024). Notoginsenoside R1 has been isolated from the roots of *Panax notoginseng* and belongs to the family of triterpenoid saponins (Huang et al., 2024; Zhang H. et al., 2024). The aforementioned study demonstrated that R1 decreased the attachment of pericytes to endothelial cells, reduced the overexpression of angiopoietin-I (Ang I) in pericytes, and inhibited downstream PI3K and AKT phosphorylation; presumably, these results are attributable to reduced angiogenesis and increased plaque stability mediated through the PI3K/AKT pathway (Li Y. et al., 2024). Another study identified that R1, ginsenoside Rg1 (Rg1), and protocatechuic aldehyde (PCAD) can act synergistically in HUVECs subjected to low shear stress (L-FSS) to modulate the expression of PI3K and AKT, activate eNOS, and increase NO production, thereby facilitating the restoration of damaged endothelium and enhancing vascular activity (Zhang L. et al., 2020). A study also found that the *Paeonia* root extract, paeonol, could activate the PI3K signaling pathway. This was evidenced by the oxidative modification of ox-LDL-induced vascular smooth muscle cell damage. The study also suggests that paeonol upregulates the expression of the autophagy-related protein Beclin-1, downregulates the expression of cysteine-requiring aspartate protease 3 (caspase-3) and sequestosome-1 (P62), and reduces the effect of apoptosis on endothelial cells by increasing the autophagy of damaged cells (Liu et al., 2021; Vellasamy et al., 2021; Wu et al., 2021). Geniposide is an iridoid glycoside extracted from *Gardenia jasminoides* (Gao and Feng, 2022; Li D. et al., 2024). Lin et al. (2024) revealed that geniposide attenuates inflammatory responses, enhances lipid metabolism, promotes autophagic activity, and suppresses foam cell formation by inhibiting the poly (ADP-ribose) polymerase 1 (PARP1)/PI3K/AKT signaling pathway. This mechanism contributes to its protective role against AS (Lin et al., 2024). Artemisinin (ART), a sesquiterpene lactone compound isolated from *Artemisia annua*, has long been the primary treatment for malaria (Ma et al., 2020; Shi et al., 2022; Yin et al., 2025). Wang P. et al. (2021) suggested that, beyond its antimalarial effects, ART also has antioxidant, anti-inflammatory, and apoptosis-inhibitory properties. They validated in hydrogen peroxide (H_2_O_2_)-stimulated endothelial cells that ART induces PI3K and AKT phosphorylation, increases the activities of eNOS and superoxide dismutase (SOD), and reduces the expressions of caspase-3 and BCL2-associated X Protein (Bax), thereby improving endothelial oxidative damage and vascular dilation dysfunction (Wang P. et al., 2021).

Gypenoside, a triterpenoid saponin isolated from *Gynostemma pentaphyllum*, is classified as both a terpenoid and a saponin compound. Initial investigations indicated that gypenoside exhibits anti-inflammatory, lipid-lowering, and antioxidant effects and provides protective effects on HUVECs (Su et al., 2021; Xie et al., 2024). Song et al. (2020) revealed that gypenoside markedly suppresses the production of key apoptosis-associated proteins, including cysteine-requiring aspartate protease 9 (caspase-9), cytochrome C (Cyt-c), caspase-3, and acute phase reaction protein (APRP), through modulation of the PI3K/AKT axis, ultimately leading to the attenuation of apoptotic processes. Furthermore, in additional mechanistic studies, gypenoside was found to help human umbilical vein cell hybrid (EA.hy926) cells resist ox-LDL-induced damage by downregulating the expression of the mitochondrial dynamics-related proteins, dynamin-related protein 1 (DRP1) and mitofusin 2 (Mfn2), and by reducing aortic mitochondrial fission (Song et al., 2020). Tanshinone IIA (TIIA), the principal bioactive compound derived from Danshen (*Salvia miltiorrhiza*), enhances the transcriptional activity of transforming growth factor-β (TGF-β) and stimulates phosphorylation of the PI3K signaling pathway. Through the TGF-β/PI3K/AKT/eNOS cascade, TIIA reduces serum concentrations of IL-6, TNF-α, and endothelin-1 (ET-1), improving the inflammatory state in blood vessels. Moreover, studies have demonstrated that TIIA suppresses protein levels, including hypoxia-inducible factor 1-alpha (HIF-1α), vascular endothelial growth factor (VEGF), and matrix metalloproteinase-9 (MMP-9), thereby inhibiting angiogenesis associated with AS (Guo et al., 2020; Wang et al., 2020; Yang et al., 2023). Geraniol (GNL), a monoterpenoid alcohol found in plants such as rose, geranium, and mint, exhibits anti-inflammatory, antioxidant, and anticancer properties (Ben Ammar, 2023; Lei et al., 2019). Research indicates that GNL pretreatment of HUVECs boosts SOD and catalase expression, increases PIK3/AKT phosphorylation, and decreases ROS, suggesting that GNL activates the PIK3/AKT protective pathway against oxidative stress in endothelial cells (Ben Ammar et al., 2022). Zedoarondiol, a sesquiterpene alcohol from *Zingiber zerumbet*, also inhibits MCP-1-induced THP-1 monocyte migration and endothelial cell adhesion and may do so by inhibiting the C-X-C motif chemokine ligand 12/C-X-C motif chemokine receptor 4 (CXCL12/CXCR4) signaling pathway, resulting in the downregulation of PI3K, AKT, and NF-kB (Chai et al., 2022). Kirenol (KRL), a principal diterpenoid component found in *Andrographis paniculata*, has been shown to protect endothelial cells against oxidative stress induced by benzo[a]pyrene (B [a]P) exposure. This protective effect is presumably mediated through the activation of the PIK3/AKT pathway, followed by the upregulation of NRF2 signaling. The process assists in clearing excess ROS while facilitating the breakdown of heme into biliverdin and carbon monoxide (Nasir et al., 2022; Rajendran et al., 2021). Astragaloside IV (AST IV) is mainly derived from *Astragalus membranaceus*. It is a triterpenoid saponin with multiple therapeutic antineoplastic, anti-inflammatory, immunoregulatory, and antioxidant activities. AST IV effectively inhibited the phosphorylation of PI3K, AKT, and mTOR in atherosclerosis mouse models, alleviated inflammatory responses, and decreased the expression of adhesion molecules, thereby demonstrating its potential as a therapeutic candidate for AS (Sun et al., 2024).

### 4.2 Alkaloids

Natural alkaloids are nitrogen-containing compounds produced by plants. Their modification by various enzymes results in diverse alkaline properties and binding capacities for biomembrane receptors (Bhambhani et al., 2021; Hashimoto and Yamada, 2003). Intimal thickening is strongly associated with the initiation of AS. In AS mice, berberine (BBR), an isoquinoline alkaloid from *Coptis chinensis*, decreases Beclin-1 and P62 expressions to modulate autophagy and inhibits PI3K and mTOR phosphorylation, resulting in dose-dependent suppression of neointimal thickening in the carotid artery (Cizek et al., 2007; Song and Chen, 2021). Nuciferine, an aporphine alkaloid naturally present in lotus leaf extracts, has been shown to downregulate the production of calmodulin 4 (Calm4), matrix metalloproteinase 12 (MMP12), and phosphorylated AKT (P-AKT) in mouse origin vascular smooth muscle (MOVAS) cells stimulated with 3% fetal bovine serum (FBS). This downregulation contributes to the inhibition of excessive proliferation and migration in vascular smooth muscle cells. Subsequent experiments confirmed that this result could be reversed by the AKT inhibitor selective AKT inhibitor (MK2206), indicating that the PI3K/AKT signaling pathway plays a crucial role in nuciferine treatment of 3% FBS-induced MOVAS (Xiao M. et al., 2023). Ferroptosis represents a unique form of programmed cell demise marked by dysregulated iron deposition and impaired lipid peroxide homeostasis. Research has shown that upregulating the expression of regeneration family member 1α (REG1A) leads to an increase in Fe^2+^ concentrations, malondialdehyde (MDA), ROS, and other lipid peroxides, while decreasing GSH and glutathione peroxidase 4 (GPX4), thereby exacerbating ferroptosis (Zhao et al., 2025). Matrine is primarily derived from *Sophora flavescens* and belongs to the quinolizidine alkaloid class. Zhao et al. (2025) demonstrated that matrine targets the REG1A protein, elevates the phosphorylation states of PI3K, AKT, and mTOR, and inhibits ox-LDL-induced endothelial cell ferroptosis. However, these effects were blocked when the PI3K inhibitor phosphoinositide 3-kinase inhibitor (LY294002) was added, suggesting that matrine alleviates AS by stimulating the PI3K/AKT/mTOR signaling cascade.

### 4.3 Flavonoids

Flavonoids are found in nearly all plant species and are the most widely distributed polyphenolic compounds. The characteristic benzopyrone skeleton and abundant phenolic hydroxyl groups confer potent antioxidant activity to flavonoids, enabling them to directly scavenge ROS or indirectly activate antioxidant-related signaling pathways, thereby protecting endothelial cells (Bondonno et al., 2024; Dobrzynska et al., 2020). Kaempferol is a natural flavonol widely found in plants and has various pharmacological activities. In human aortic endothelial cells (HAECs) stimulated with ox-LDL, G protein-coupled estrogen receptor (GPER) expression was significantly increased, and the P-AKT/AKT ratio was upregulated, while TNF-α and IL-6 levels in the serum were reduced. Additionally, under the influence of kaempferol, lipid levels (TG and TC), the Bax/Bcl-2 ratio related to apoptosis, and caspase-3 expression all exhibited a similar decreasing trend. However, most of these effects were reversed after si-GPER transfection, indicating that kaempferol alleviates AS through the upregulation of GPER expression and subsequent activation of the PI3K/AKT signaling cascade (Feng et al., 2021). Rutin, a bioflavonoid extracted from the leaves of *Ginkgo biloba*, exhibits lipophilic characteristics and displays multiple pharmacological effects, including antimicrobial, antioxidant, and anti-inflammatory effects (Liu H. et al., 2024). A study by Li et al. revealed that Rutin enhances Arg-1 (Arginase-1) expression while decreasing MCP-1, IL-1β, and inducible nitric oxide synthase (iNOS) levels, thereby facilitating macrophage polarization toward the M2 phenotype. Furthermore, they found that Rutin, by inhibiting the PI3K/AKT/mTOR axis, induces the fusion and degradation of autophagosome-lysosome in macrophages, enhances cellular autophagy, and reduces foam cell formation (Li et al., 2022). Morin hydrate is a flavonol extracted from plants such as *Allium cepa* and *Psidium guajava*. It has been reported that morin hydrate (MO) downregulates the phosphorylation levels of nuclear factor kappa B subunit P65 (p65), inhibitor of kappa B α (IκBα), PI3K, and AKT in a time- and dose-dependent manner, inhibiting the PI3K/AKT and NF-κB-related signaling pathways, reducing the expression of inflammatory factors such as VCAM-1, ICAM-1, MMP-9, and cyclooxygenase-2 (COX-2), thereby exerting protective effects on HUVECs (Meng et al., 2021; Rajput et al., 2021). Lymphatic circulation is a key pathway for promoting reverse cholesterol transport (RCT). Nevertheless, during the advanced phases of AS, under the stimulation of inflammatory cells, the dilation and increased density of lymphatic vessels in the arterial wall significantly affect RCT, leading to the accumulation of lipid and cholesterol crystals and further aggravating atherosclerotic plaque development (Brakenhielm et al., 2024). Lymphatic vessel formation is mainly initiated by vascular endothelial growth factor C (VEGF-C), which is secreted by macrophages. It has been reported that a chalcone flavonoid compound derived from safflower, hydroxysafflor yellow A, inhibits the activity of phosphatidylinositol-4, 5-bisphosphate 3-kinase catalytic subunit alpha (PI3Kα) by binding to its specific binding domain, downregulating the levels of TNF-α, IL-6, and MCP-1, and further reducing the expressions of P-AKT, P-mTOR, and VEGF-C. This suggests that inhibition of the PI3K/AKT signaling pathway effectively prevents lymphangiogenesis and the inflammatory response in the treatment of AS (Feng et al., 2023).

### 4.4 Polysaccharides

Polysaccharides represent a class of high-molecular-weight biopolymers formed through glycosidic linkages connecting numerous monosaccharide units. They are essential for life and widely distributed in nature, including in plants, animals, and algae (Yu et al., 2018). Its bioactivity is determined by the linkage pattern of glycosidic bonds and the spatial conformation. For instance, galactomannan exhibits high viscosity due to its linear structure, which contributes to its cholesterol-lowering effect (Silva et al., 2021). *Lycium barbarum* polysaccharide (LBP) constitutes the principal bioactive component derived from *Lycium barbarum* (Zhou et al., 2022). LBP dose-dependently increases the ratio of the cell cycle G1/G2, upregulates the contraction phenotype marker (α-smooth muscle actin, α-SMA), downregulates the synthetic phenotype marker OPN, inhibits homocysteine (Hcy)-induced proliferation of vascular smooth muscle cells (VSMCs), and facilitates their shift toward a contractile phenotype. Meanwhile, LBP has been shown to inhibit the expressions of PI3K and AKT proteins, upregulate the expression of the anti-proliferative gene *microRNA-145*, and increase α-SMA levels, suggesting that LBP exerts its anti-AS effect through the PI3K/AKT signaling pathway (Zhang M. et al., 2020). Tea polysaccharide (TPS3A) is a polysaccharide derived from tea leaves that exhibits notable anti-dyslipidemic and anti-AS effects. TPS3A plays a cytoprotective role in human liver cancer (HepG2) cells under insulin resistance. It regulates insulin-triggered increases in acetyl-CoA carboxylase 1 (ACC1), SREBP-1, and apolipoprotein B (apoB) and prevents the buildup of intracellular lipids. In the presence of PI3K or AKT inhibitors, the protective effects of TPS3A were amplified, with TG, free fatty acids (FFAs), and LDL levels being significantly reduced in HepG2 cells. This suggests the importance of the PI3K/AKT signaling pathway in the mechanism through which TPS3A reduces aberrant hepatic LDL. This, in turn, helps combat AS (Kuang et al., 2025). Konjac glucomannan (KGM) is a type of water-soluble dietary fiber. It is a polysaccharide that consists of polymerized glucose and mannose units. The activation of PI3K/AKT signaling is responsible for reducing foam cell formation, increasing the aortic lumen diameter, and reducing the size of atherosclerotic plaques in animal models with AS. At its core, the mechanism appears to involve the inhibition of MDA and myeloperoxidase (MPO), a reduction in the production of C-reactive protein (CRP), TNF-α, and IL-6, and decreased levels of TC, TG, and LDL-C. These effects collectively help alleviate AS, primarily by boosting antioxidant defenses, tamping down inflammatory reactions, and rebalancing lipid metabolism (Weng et al., 2023).

### 4.5 Stilbenoid compounds

Stilbenoids are a group of low-molecular-weight phenolic compounds. The hydroxyl groups attached to the phenyl ring are responsible for its anti-inflammatory properties, its modulatory effects on glucose and lipid metabolism, and other biological activities relevant to anti-AS effects (Kaur et al., 2024). Resveratrol is a polyphenolic compound that was first isolated from *Veratrum grandiflorum* and is abundantly found in grapes, wine, peanuts, soybeans, and various berries (Breuss et al., 2019). Ji et al. (2022) found that resveratrol (RV) can lower serum TNF-α and CRP levels, inhibit the expressions of MMP-9 and CD40 ligand (CD40L) in affected tissues, and reduce both acute and chronic inflammatory responses in AS mice. Furthermore, RV significantly inhibits weight gain in mice, downregulates serum LDL, TG, and TC levels, and improves lipid profiles. Liver dysfunction exacerbates the progression of AS. RV has been found to inhibit the activity of 3-hydroxy-3-methylglutaryl-CoA (HMG-CoA) reductase and reduce liver cell injury markers such as alanine aminotransferase (ALT), aspartate aminotransferase (AST), and alkaline phosphatase (ALP), all of which are associated with the PI3K/AKT signaling pathway (Ji et al., 2022). Glycolysis is an essential metabolic reprogramming event in AS-affected cells that triggers mitochondrial membrane permeabilization and the activation of apoptosis-related proteins Bax and Bak, thereby reducing plaque stability. RV alleviates AS by activating the PI3K/AKT signaling pathway and targeting key enzymes in the glycolytic pathway, such as GLUT1, hexokinase 2 (HK2), and fructose-2, 6-bisphosphatase 3 (PFKFB3), thereby inhibiting glycolysis (Jiang et al., 2022; Pan et al., 2025). 2,3,5,4′-Tetrahydroxystilbene 2-O-β-D-glucoside (TSG), a glycosylated derivative of resveratrol obtained from *Polygonum multiflorum* (He Shou Wu), exhibits marked anti-atherosclerotic properties (Zhang et al., 2008). TSG can reduce plaque area in high-fat-diet-induced AS mice, lower serum lipid levels and inflammatory factor concentrations, and decrease the expression of autophagy-related proteins microtubule-associated protein 1A/1B-light chain 3 (LC3I) and P62 (Yang et al., 2024). *In vitro*, TSG suppresses dendritic cell (DC) maturation, facilitates the induction of regulatory T cells (Treg) from T lymphocytes, and disrupts lipid deposition in DCs induced by ox-LDL, thereby preserving intracellular lipid homeostasis. These observed outcomes are strongly associated with the inhibition of the PI3K/AKT signaling cascade (Yang et al., 2024).

### 4.6 Phenylpropanoids

Phenylpropanoids constitute a class of natural products characterized by a C_6_–C_3_ carbon skeleton. These molecules possess multiple bioactive properties, including anti-inflammatory, anticancer, neuroprotective, and antioxidative effects (Rodrigues et al., 2024). Urolithin B (Uro B) is a metabolic derivative of plants such as pomegranate and walnut. It has been shown to inhibit PI3K and AKT phosphorylation, upregulate the expression of α-SMA and smooth muscle 22α protein (SM22α), promote the platelet-derived growth factor BB (PDGF-BB) dimer-induced conversion of smooth muscle cells to a contractile phenotype, and restrain excessive migration and proliferation of these cells, thereby delaying intimal thickening and plaque growth in AS (Li et al., 2025; Piwowarski et al., 2015). Myristicin is a natural phenylpropene ether isolated from nutmeg. Luo et al. (2022) demonstrated that myristicin can reduce the release of MCP-1, ICAM-1, VCAM-1, and IL-6 in HUVECs exposed to ox-LDL and decrease the expression of Bax and MMP-9 genes. Additionally, similar trends were observed in ox-LDL-stimulated human vascular smooth muscle cells (HVSMCs). These effects were shown to be associated with myristicin-induced inactivation of the PI3K/AKT/NF-κB signaling cascade. Schisandrin C (SC) is a lignan compound found in Schisandra. It negatively regulates the PI3K/AKT/mTOR axis, increases the LC3 II/LC3 I ratio and Beclin-1 expression in ox-LDL-induced HUVECs, and inhibits P62 protein accumulation, thereby improving autophagy and protecting endothelial cells (Duan et al., 2024).

### 4.7 Others

**FIGURE 3:**
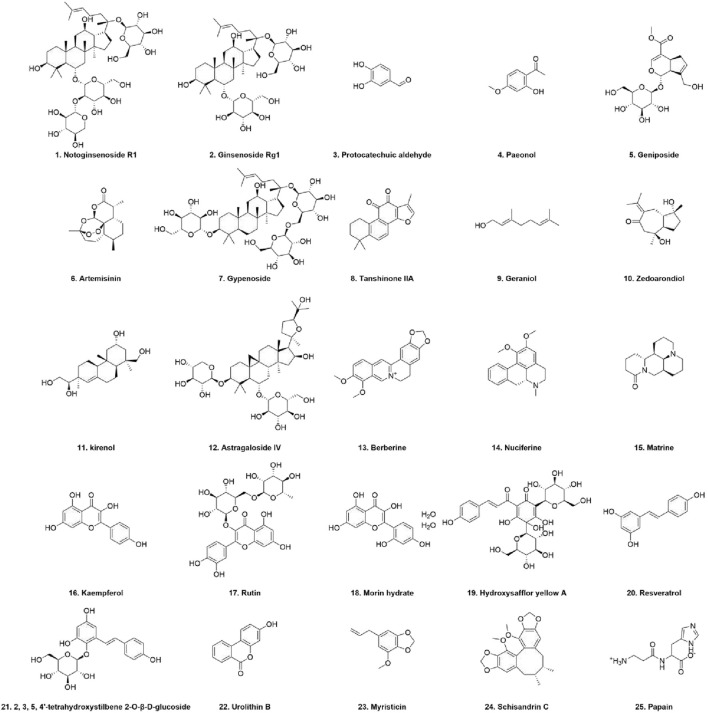
Chemical structural formula of plant-derived compounds.

Withaferin-A (WA) is a steroidal compound found in a variety of Solanaceae plants, such as Datura, Withania, and Lycium (Sultana et al., 2021). Studies have shown that WA administration decreases the levels of the major lipid peroxidation indicators, such as thiobarbituric acid reactive substances (TBARSs), MPO, and CRP, in rats that have undergone AS development due to a high-cholesterol diet. In addition, WA decreases caspase-3 and Bax protein expression while increasing GSH and enhancing the activity of critical antioxidant enzymes, such as GPX, SOD, and CAT, thereby alleviating AS through anti-apoptosis, antioxidant, and ferroptosis-inhibitory effects. WA not only affects apoptosis and systemic antioxidative capacity but also exerts vaso- and lipido-regulatory properties, reducing serum inflammatory markers and blood lipid levels (Zhang L. et al., 2022). It also suppresses intracellular cyclooxygenase-2 (COX-2) expression, contributing to its broader antioxidant, anti-inflammatory, and anticoagulant activities (Fei et al., 2018; Simion et al., 2020). Additionally, papain has been shown to downregulate MCP-1 and prostaglandin E2 (PGE2) expressions, reduce mitogen-activated protein kinase 14 (P38) and p65 levels, and inhibit the PI3K/AKT–NF-κB axis and the mitogen-activated protein kinase (MAPK) cascade. All of them would limit foam cell formation and, to a certain extent, prevent lipid deposition (Fei et al., 2023). The natural peptide tartary buckwheat protein-derived peptide (AFYRW), derived from the protein of *Fagopyrum tataricum*, was able to block PI3K and AKT phosphorylation, prevent NF-κB nuclear translocation, and downregulate P65 expression. It also reduced ROS, MDA, and VEGF levels while antagonizing H_2_O_2_-induced abnormal proliferation and oxidative stress in HUVECs (Xiao Y. et al., 2023) (Figure 3 and Tables 1, 2).

**TABLE 1:** Mechanism of action of phytochemicals to regulate the PI3K/AKT signaling pathway to improve atherosclerosis.

Compound	Animal/cells	Dose/concentration	Effect	Upregulation	Downregulation	*In vitro*/*in vivo*
Notoginsenoside R1​	HFD-induced atherosclerosis in ApoE−/− mice; VEGF-A-induced HUVECs and VEGF-A-induced HBVPs	10 mg/(kg·d); 400 μg/mL	Improves lipid metabolism; inhibits angiogenesis	α-SMA; PDGFR-β	TC; TG; LDL-C; Ang-1; Tie2; p-PI3K; p-AKT; CD31; VE-cadherin; and integrin-β1	*In vitro*/*in vivo*
Ginsenoside Rg1–notoginsenoside R1–protocatechuic aldehyde	HFD-induced atherosclerosis in ApoE−/− mice; L-FSS-induced HUVECs	[10 mg Rg1 + 10 mg R1 + 14 mg PCAD]/(kg·d); (100 μg Rg1 + 100 μg R1 +30 μg PCAD)/mL	Improves lipid metabolism; suppresses cell proliferation; inhibits cell migration; and promotes vasodilation	eNOS; PGI2; PI3K; AKT; HDL-C; and FAK	TC; TG; LDL-C; ET-1; and TXA2	*In vitro*/*in vivo*
Paeonol	HFD-induced atherosclerosis in ApoE−/− mice; ox-LDL-induced VSMCs	400, 200, and 100 mg/(kg·d); 30, 60, and 120 μmol/L	Enhances cellular autophagy; inhibits apoptosis	LC3II; PI3K; and Beclin-1	Caspase-3; P62	*In vitro*/*in vivo*
Geniposide​	HFD-induced atherosclerosis in ApoE−/− mice; ox-LDL-induced VSMCs	12.5, 25, and 50 mg/kg; 50, 100, and 500 μM	Improves lipid metabolism; attenuates vascular inflammation; suppresses foam cell formation; and enhances cellular autophagy	HDL-C; IL10; LAMP1; and ABCG1	TC; TG; LDL-C; IL6, TNF-α, CD36; P-PI3K; P-AKT; P62; and PARP1	*In vitro*/*in vivo*
​Artemisinin​	H2O2-induced HUVECs	3, 10, 30, and 100 μM	Inhibits apoptosis; inhibits oxidative stress; and promotes vasodilation	NO; P-AKT; P-eNOS; Bcl-2; and SOD	ROS; Caspase-3; Bax; and MDA	*In vitro*
​Gypenoside	HFD-induced atherosclerosis in ApoE−/− mice; ox-LDL-induced EA. hy926 cells	2.973 mg/(kg·d); 100 μg/mL	Improve lipid metabolism; inhibit apoptosis	PI3K; P-AKT; and P-Bad	TC; TG; LDL-C; PARP; Cyt-c; caspase-9; caspase-3; Mfn2; Atp12a; Cox5a; Sdhc; Ndufb6; and DRP1	*In vitro*/*in* *vivo*
Tanshinone IIA	HFD-induced atherosclerosis in ApoE−/− mice	10 mg/(kg·d)	Improves lipid metabolism; attenuates vascular inflammation; and inhibits angiogenesis	HDL-C; NO; PI3K; p-eNOS; p-AKT; and TGF-β	TC; TG; LDL-C; TNF-α; IL-6; ET-1; MMP-9; VEGF; and HIF1-α	*In vivo*
Geraniol	Ox-LDL-induced HUVECs	50 and 100 μM/mL	Improves lipid metabolism; attenuates vascular inflammation; inhibits oxidative stress; and inhibits apoptosis	Nrf2; HO-1; NQO-1; γ-GCLC; P-PI3K; P-AKT; and HO-1	Ox-LDL; TC; ROS; TNF-α; IL-6; IL-1β; TGF-β; κVCAM-1; ICAM-1; FABP4; MDA; IB-α; NF-κB; and P65	*In vitro*
Zedoarondiol	HFD-induced atherosclerosis in ApoE−/− mice; CXCL12 and CCL2-induced THP-1 cells; ox-LDL-induced HUVECs	20 mg/(kg·d); 10, 20, and 40 μg/mL; 10, 20, and 40 μg/mL	Improves lipid metabolism; attenuates vascular inflammation; inhibits monocyte migration; and inhibits monocyte adhesion	HDL-C	TC; TG; LDL-C; MCP-1; IL-1β; TNF-α; VCAM-1; ICAM-1; CXCL12; CXCR4; P13K; AKT; and NF-κB	*In vitro*/*in vivo*
Kirenol	B [a]P-induced HUVECs	5, 10, and 25 μmol	Inhibits oxidative stress; inhibits apoptosis	NO; P-PI3K; P-AKT; Bcl-2; NQO-1; HO-1; and Nrf2	ROS; 4-HNE; and caspase-3	*In vitro*
Astragaloside IV	WD-induced atherosclerosis in SD rats	20 mg/(kg·d)	Improves lipid metabolism; modulates gut microbiota; attenuates vascular inflammation; and inhibits monocyte adhesion	HDL-C	TC; TG; LDL-C; ET-1; Ang-II; TNF-α; IL-6; IL-1β; VCAM-1; MMP-2; MCP1; ICAM-1; p-PI3K; p-AKT; and p-mTOR	*In vivo*
Berberine	HFD-induced atherosclerosis in ApoE−/− mice	78, 117, and 156 mg/kg	Improves lipid metabolism; inhibits apoptosis	HDL-C	TC; TG; LDL-C; Beclin-1; P62; p-AKT; p-PI3K; and p-mTOR	*In vivo*
Nuciferine	HFD-induced atherosclerosis in ApoE−/− mice; FBS-induced MOVAS	5, 20, and 40 mg/(kg·d); 1, 5, and 10 μM	Suppresses aberrant cell proliferation; inhibits cell migration	NS	Calm4; α-SMA-PCNA; MMP12; and p-AKT	*In vitro*/*in vivo*
Matrine	Ox-LDL-induced HUVECs	1, 2, and 3 mg/mL	Improves lipid metabolism; inhibits ferroptosis	HDL-C; GSH; GPX4; SLC7A11; PI3K; AKT; and mTOR	TC; TG; LDL-C; Fe^2+^; MDA; and REG1A	*In vitro*
Kaempferol	OVX + HFD-induced atherosclerosis in ApoE−/− mice; ox-LDL-induced HAECs	50 and 100 mg/(kg·d); 5, 10, and 20 μg/mL	Improves lipid metabolism; attenuates vascular inflammation; inhibits oxidative stress; and inhibits apoptosis	HDL-C; SOD; GSH; GPER; PI3K; AKT; Nrf2; and HO-1	TC; TG; LDL-C; TNF-α; IL-6; ICAM; VCAM; caspase-3; Bax/Bcl-2; and ROS	*In vitro*/*in vivo*
Rutin	Ox-LDL-induced RAW264.7 cells	12. 5 μg/mL	Attenuates vascular inflammation; enhances macrophage autophagy; suppresses foam cell formation; and promotes M2 macrophage polarization	Arg1; LC3II	TC; TG; LDL-C; iNOS; Mcp1; ROS; p62; PI3K; AKT; mTOR; IL-1β; and LC3I	*In vivo*
Morin hydrate	HFD-induced atherosclerosis in ApoE−/− mice; LPS-induced HUVECs	50 mg/(kg·d); 16.5, 33, and 66 μM	Enhances macrophage autophagy; improves lipid metabolism; and attenuates vascular inflammation	HDL-C; LC3II	TC; TG; LDL-C; VCAM-1; CAM-1; P62; COX-2; MMP-9; TNF-α; IL-6; THP-1; PI3K; p-AKT1; p-p65; and p-IκBα	*In vitro*/*in vivo*
Hydroxysafflor yellow A	HFD-induced atherosclerosis in ApoE−/− mice; LPS- and ox-LDL-induced RAW264.7 cells	6.25, 12.5, and 25 mg/kg (4/w); 150 μM and 300 μM	Improves lipid metabolism; attenuates vascular inflammation; and reduces lymphangiogenesis	HDL-C	TC; TG; LDL-C; TNF-α; IL-6; MCP-1; VEGF-C; PI3K; AKT; and mTOR	*In vitro*/*in vivo*
*Lycium barbarum* polysaccharide	Hcy-induced VSMCs	1, 2, and 4 g/mL	Suppresses aberrant cell proliferation; induces cell cycle transition from G2 to G1; and promotes phenotypic switch of vascular smooth muscle cells to a contractile state	α-SMA; SM22α; miR-145; and G2	OPN; PI3K; AKT; and G1	*In vitro*
Tea polysaccharide	HFD-induced atherosclerosis in ApoE−/− mice; absence of 0. 1 μM insulin-induced HepG2 cells	100 and 300 mg/(kg·d); 5 and 50 μg/mL	Attenuates vascular inflammation; inhibits oxidative stress; and improves lipid metabolism	IL-10; HDL	TNF-α; IL-1β; IL-6; MDA; VLDL; CETP; TG; apoB; apoCIII; MTP; SREBP-1; ACC1; FAS; P-PI3K; P-AKT; P-mTORC1; and P-Fox01	*In vitro*/*in vivo*
*Konjac glucomannan*	HFD-induced atherosclerosis in rabbits	300 mg/(kg·d)	Improves lipid metabolism; attenuates vascular inflammation; improves endothelial function; and inhibits oxidative stress	HDL-C; GSH-px; SOD; P-AKT; P-PI3K; and NO	TC; TG; LDL-C; VCAM-1; ET; MPO; and MDA	*In vivo*
Resveratrol	HFD-induced atherosclerosis in ApoE−/− mice; PI3K inhibitor-induced HUVECS	50 mg/(kg·d); 1 mg/mL	Improves lipid metabolism; attenuates vascular inflammation	NS	TC; TG; LDL-C; HDL-C; TNF-α; CRP; MMP-9; CD40L; HMG-CoA; LDH; CPK; PI3K; AKT; and mTOR	*In vitro*/*in vivo*
Resveratrol	Carotid balloon injury-induced atherosclerosis in SD rats; ox-LDL-induced HUVECs	10 and 50 mg/(kg·d); 80 μM/L	Improves lipid metabolism; inhibits cellular glycolysis; and inhibits apoptosis	Bcl-2; HDL-C; and P-AKT	TC; TG; LDL-C; GLUT1; HK2; PFKFB3; Bax; Caspase-3; and LA	*In vitro*/*in vivo*
2, 3, 5, 4′-Tetrahydroxystilbene 2-O-β-D-glucoside ​	HFD-induced atherosclerosis in ApoE−/− mice; ox-LDL-induced BMDCs	40 mg/(kg·d); 40 and 80 μM	Improves lipid metabolism; enhances cellular autophagy; and attenuates vascular inflammation	Treg	TC; TG; LDL-C; PI3K; p-AKT; p-mTOR; CD80; CD86; MHC-II; Th17; LC3I; and P62	*In vitro*/*in vivo*
Urolithin B	PDGF-BB-induced VSMCs	40 μM	Suppresses cell proliferation; inhibits cell migration; and promotes phenotypic switch of vascular smooth muscle cells to a contractile state	α-SMA; SM22α	OPN; P-AKT	*In vitro*
Myristicin	Ox-LDL-induced HVSMCs and HUVECs	5, 25, and 50 μM	Inhibits apoptosis; attenuates vascular inflammation; inhibits cell migration; and suppresses cell proliferation	Bcl-2	Bax; MMP-9; MCP-1; IL-6; VCAM-1; ICAM-1; p-AKT; and P65	*In vitro*
Schisandrin C	Ox-LDL-induced HUVECs	1, 5, and 25 μmol	Attenuates vascular inflammation; enhances cellular autophagy	Beclin1; ATG5; and LC3II	TNF-α; IL-1β; P-PI3K; P-Akt; P-mTOR; P62; and LC3I	*In vitro*
Withaferin-A	HCD-induced SD rats	10 mg/(kg·d)	Improves lipid metabolism; inhibits oxidative stress; attenuates vascular inflammation; and inhibits apoptosis	HDL-C; SOD; CAT; GPx; GSH; Bcl-2; PI3K; and AKT	TC; TG; LDL-C; TBARS; MPO; CRP; ​**​**Ceruloplasmin​​; WBC; COX; 15-LOX; PGE2; TNF-α; IL-6; iNOS; NO; Bax; Fas; Bim; caspase-3; and caspase-9	*In vivo*
Papain	HFD-induced atherosclerosis in Wistar rats; thrombin-induced co-culture THP-1 cells with platelets	100, 200, and 400 U/L/d; 200U/L	Suppresses monocyte-to-macrophage differentiation; suppresses foam cell formation; and improves lipid metabolism	NS	CD11b; CD36; TG; PGE2; MCP-1; P38; P-AKT; P-JNK; NF-κB; p65; COX2; TNF-α; IL-1; T-CHO; LDL-C; CD14; and CD41	*In vitro*/*in* *vivo*
Tartary buckwheat protein-derived peptide	H_2_O_2_-induced HUVECs	10 and 60 μg/mL	Inhibits oxidative stress; inhibits angiogenesis; and attenuates vascular inflammation	NS	NO; MDA; PI3K; AKT; ROS; VEGF; TNF-α; IL-6; VCAM-1; and NF-κB	*In vitro*

Abbreviations: HBVPs, Human brain vascular pericytes; Tie2, tyrosine kinase with immunoglobulin-like and EGF-like domains 2; VE-cadherin, vascular endothelial cadherin; integrin-β1, extracellular matrix receptor; TXA2, thromboxane A2; PARP1, poly (ADP-ribose) polymerase 1; Atp12a, ATPase H+/K+ transporting non-gastric alpha subunit; Cox5a, cytochrome c oxidase subunit 5a; Sdhc, succinate dehydrogenase complex subunit C; Ndufb6, NADH: ubiquinone oxidoreductase subunit B6; HIF1-α, hypoxia-inducible factor 1-α; G2, gap 2 phase; MMP-9, matrix metalloproteinase-9; ICAM, intercellular adhesion molecule; FABP4; IkB-α, inhibitor of nuclear factor kappa B-α; CXCL12, C-X-C motif chemokine ligand 12; CXCR4, C-X-C chemokine receptor type 4; 4-HNE, 4-hydroxynonenal; Ang-II, angiotensin II; Bcl-2, B-cell lymphoma 2; PCNA, proliferating cell nuclear antigen; Cox, cyclooxygenase; MMP-12, matrix metalloproteinase-12; VLDL, very low-density lipoprotein; CETP, cholesteryl ester transfer protein; Fe^2+^, ferrous iron; ACC1, acetyl-CoA carboxylase 1; T-CHO, total Cholesterol; CPK, creatine phosphokinase; Fox01, forkhead box protein O1; apoCIII, apolipoprotein C-III; MTP, microsomal triglyceride transfer protein; FAS, fatty acid synthase; LA, linoleic acid; MHCII, major histocompatibility complex class II; Th17, T helper cell 17; LC3, microtubule-associated protein 1A/1B-light chain 3; P-JNK, phospho-c-Jun N-terminal kinase; Arg1, arginase 1; GSG-px, glutathione peroxidase; WBC, white blood cell; 15-Cox, 15-lipoxygenase; Bim, Bcl-2-like protein 11; r-GCLC, glutamate-cysteine ligase catalytic subunit; PDGFR-β, platelet-derived growth factor receptor β; SLC7A11, solute carrier family 7 member 11; PGI2, prostacyclin; FAK, focal adhesion kinase; LC3II, lipidated form of LC3; LAMP1, lysosomal-associated membrane protein 1; ABCG1, ATP-binding cassette sub-family G member 1; Bad, Bcl-2-associated death promoter; NS, not significant.

**TABLE 2:** Fundamental information on phytochemicals.

Compound	Type	Species name	Authority	Family	Genus
Notoginsenoside R1	Triterpenoid glycoside	*Panax notoginseng*	F.H.Chen	Araliaceae	Panax
Ginsenoside Rg1	Triterpenoid glycoside	*Panax ginseng*	C.A.Mey	Araliaceae	Panax
Protocatechuic aldehyde	Phenolic aldehyde	*Hordeum vulgare*	L	Poaceae	Hordeum
Paeonol	Monoterpenoid phenol	*Paeonia suffruticosa*	Andrews	Paeoniaceae	Paeonia
Geniposide	Iridoid glycosides	*Gardenia jasminoides*	J.Ellis	Rubiaceae	Gardenia
Artemisinin	Sesquiterpene lactone	*Artemisia annua*	L	Asteraceae	Artemisia
Gypenoside	Triterpenoid saponin	*Gynostemma pentaphyllum*	Makino	Cucurbitaceae	Gynostemma
Tanshinone IIA	Diterpenoid quinone	*Salvia miltiorrhiza*	Bunge	Lamiaceae	Salvia
Geraniol	Monoterpenoid alcohol	*Guatteria ucayalina*	Huber	Annonaceae	Guatteria
Zedoarondiol	Sesquiterpene alcohol	*Zingiber zerumbet*	Roscoe ex Sm	Zingiberaceae	Zingiber
Kirenol	Diterpenoid	*Andrographis paniculata*	Wall. ex Nees	Acanthaceae	Andrographis
Astragaloside IV	Triterpenoid saponin	*Astragalus mongholicus*	Bunge	Fabaceae	Astragalus
Berberine	Isoquinoline alkaloid	*Coptis chinensis*	Franch	Ranunculaceae	Coptis
Nuciferine	Aporphine alkaloid	*Nelumbo nucifera*	Gaertn	Nelumbonaceae	Nelumbo
Matrine	Quinolizidine alkaloid	*Sophora flavescens*	Aiton	Fabaceae	Sophora
Kaempferol	Flavonol	*Kaempferia galanga*	L	Zingiberaceae	Kaempferia
Rutin	Flavonoid glycoside	*Ginkgo biloba*	L	Ginkgoaceae	Ginkgo
Morin hydrate	Flavonol	*Allium cepa*	L	Amaryllidaceae	Allium
Hydroxysafflor yellow A	Chalcone flavonoid	*Carthamus tinctorius*	L	Asteraceae	Carthamus
*Lycium barbarum* polysaccharide	Polysaccharide	*Lycium barbarum*	L	Solanaceae	Lycium
Tea polysaccharide	Polysaccharide	*Camellia sinensis*	Kuntze	Theaceae	Camellia
Konjac glucomannan	Polysaccharide	*Amorphophallus konjac*	K. Koch	Araceae	Amorphophallus
Resveratrol	Stilbenoid	*Veratrum grandiflorum*	O.Loes	Melanthiaceae	Veratrum
2, 3, 5, 4′-Tetrahydroxystilbene 2-O-β-D-glucoside	Stilbene glycoside	*Pleuropterus multiflorus*	Turcz. ex Nakai	Polygonaceae	Pleuropterus
Urolithin B	Benzopyranone derivative	*Punica granatum*	L	Lythraceae	Punica
Myristicin	Phenylpropene	*Myristica fragrans*	Houtt	Myristicaceae	Myristica
Schisandrin C	Lignan	*Schisandra bicolor*	W.C.Cheng	Schisandraceae	Schisandra
Withaferin-A	Withanolide	*Withania somnifera*	Dunal	Solanaceae	Withania
Papain	Proteolytic enzymes	*Carica papaya*	L	Caricaceae	Carica
Tartary buckwheat protein-derived peptide	Polypeptide	*Fagopyrum tataricum*	Gaertn	Polygonaceae	Fagopyrum

## 5 Consideration of PAINS properties in plant-derived compounds

Pan-assay interference compounds (PAINS) represent a class of molecules frequently associated with false-positive outcomes in high-throughput screening assays. These compounds commonly feature electrophilic groups that enable covalent conjugation with nucleophilic amino acid side chains on proteins. Conversely, they may self-assemble into colloidal aggregates in aqueous solutions, leading to non-specific protein adsorption and co-precipitation. Such interactions, however, are typically non-specific and can be highly misleading in mechanistic studies of bioactive compounds. Failure to discern the true mechanism behind apparent positive results may lead to significant misallocation of research resources. A review of databases such as PubMed, CNKI, and MEDLINE indicates that among the 30 studies included in this analysis, at least seven plant-derived compounds—geraniol, berberine, kaempferol, rutin, resveratrol, withaferin A, and artemisinin—have been identified as belonging to the PAINS category (Baell, 2016; Guerra et al., 2022; Magalhães et al., 2022). Nevertheless, although the PAINS attributes of these plant-derived compounds warrant careful consideration, they should not be summarily dismissed. Instead, rigorous experimental designs and computational validation approaches can be employed to confirm their specific target engagement, thereby enhancing the reliability of their purported therapeutic effects.

## 6 Perspectives and discussion

### 6.1 Mechanism summary

The PI3K/AKT signaling pathway represents a critical intracellular signal transduction cascade, intimately linked with lipid plaque accumulation, chronic inflammation, and ferroptosis (Deng and Zhou, 2023; Zhang D. et al., 2022). This study systematically reviews the effects of plant-derived compounds on AS mouse and cellular models by targeting the PI3K/AKT pathway. Experimental results demonstrate that these compounds exert significant antagonistic effects on AS progression. For instance, lipid overload serves as a key trigger for AS. Notoginsenoside R1 and rutin suppress the phosphorylation of PI3K and AKT, reduce SREBP-1 expression, and promote cholesterol efflux, thereby effectively ameliorating circulating lipid levels. Inflammation and oxidative stress act as exacerbating levers in AS. Geraniol, by modulating the PI3K/AKT axis and the Nrf2 pathway, upregulates the antioxidant enzyme SOD, while furanodiol inhibits monocyte migration, thereby protecting endothelial cells from damage induced by inflammatory and oxidative stressors. Consequently, the judicious application of plant-derived compounds may represent a promising direction for future AS therapeutic research.

A critical appraisal of current evidence reveals that plant-derived compounds targeting the PI3K/AKT signaling pathway exhibit a complex “dual-edged sword” effect in AS management. Intriguingly, both activation and suppression of this pathway by plant-derived compounds have demonstrated therapeutic efficacy against AS, yet the underlying mechanistic rationale remains inadequately elucidated. For instance, morin hydrate and myristicin attenuate the production of pro-inflammatory cytokines such as IL-6 and IL-1β by inhibiting PI3K/AKT activation, whereas TPS3A impedes cholesterol and fatty acid synthesis by suppressing PI3K phosphorylation and reducing SREBP-1 activity, thereby delaying the initiation and progression of AS. On the other hand, plant compounds such as matrine and kaempferol activate the PI3K/AKT signaling pathway, upregulating key ferroptosis-related targets including GSH and GPX4, thereby inhibiting ferroptotic cell death. Meanwhile, kirenol and geraniol enhance PI3K activity, leading to elevated expressions of HO-1 and Nrf2, which collectively ameliorate oxidative stress and help maintain plaque stability in advanced atherosclerosis. Notably, Hu et al. (2023) observed that the cGAS–STING signaling pathway exhibits a dual regulatory role in alleviating peripheral neuropathic pain, dependent on differential downstream molecular mechanisms and cell types involved. It is, therefore, plausible to speculate that such context-dependent effects may also apply to atherosclerosis in a stage-specific manner. For instance, excessive activation of this pathway in early disease stages may stimulate mTOR and NF-κB signaling, exacerbate inflammatory responses and dyslipidemia, and thereby promote plaque formation. In contrast, moderate activation in the later stages could facilitate the expression of Beclin-1, GSH, and GPX4, promote ferroptosis and autophagy in pathological cells, and enhance the stability of established plaques (Inada et al., 2008). Nevertheless, the observed effects may also be attributable to stochastic variations inherent in individual studies, which await further validation in future investigations. Furthermore, the majority of plant-derived compounds examined—such as artemisinin, geniposide, and zedoarondiol—demonstrate a marked capacity to significantly reduce serum levels of TG, TC, and LDL. This lipid-lowering activity may operate through two potential mechanisms: either via direct promotion of cholesterol efflux mediated by the PI3K/AKT signaling pathway or indirectly through attenuation of atherosclerotic progression influenced by this pathway, ultimately resulting in improved lipid profiles. However, the precise mechanism remains largely unelucidated in most existing studies. Thus, whether certain plant-derived compounds exert their hypolipidemic effects specifically through the PI3K/AKT signaling axis warrants further rigorous investigation.

### 6.2 Limitations

Plant-derived compounds demonstrate considerable potential in the amelioration of AS, yet several challenges must be addressed to facilitate their clinical translation. Preclinical studies have consistently shown that numerous plant-derived compounds reduce aortic plaque area and confer cardioprotective effects in animal models. For instance, myristicin and geraniol significantly attenuate oxidative stress, suppress inflammatory responses, and improve lipid profiles. However, the transition of these beneficial effects from animal models to human applications remains fraught with difficulties, chief among them being poor absorption, rapid metabolism, and low aqueous solubility, which collectively limit their bioavailability (Liu et al., 2025). For example, the oral bioavailability of resveratrol and curcumin is typically less than 1%, considerably constraining their therapeutic efficacy (Bijak, 2017; Yang et al., 2007). To overcome these limitations, advanced drug delivery strategies—such as chemical modification with hydrophilic groups or encapsulation into nanoparticle-based systems—represent promising avenues for enhancing their stability and absorption (Al-Kassas et al., 2017). Furthermore, safety profiling cannot be overlooked in the development of plant-based therapies. Administration of berberine has been associated with adverse effects, including diarrhea, nausea, and hypoglycemia (Asghari et al., 2025). Similarly, tanshinone IIA may elevate the risk of postpartum eclampsia in pregnant women, underscoring the necessity for rigorous toxicological evaluation and patient-specific risk assessment (Zhou Z. Y. et al., 2019). Current research findings predominantly focus on therapeutic efficacy and molecular mechanisms, yet there remains a lack of standardized and systematic evaluation regarding the toxicity and systemic side effects of plant-derived compounds in humans. We posit that establishing rigorous safety monitoring parameters in clinical trials, systematically categorizing the toxic and adverse effects of various plant-derived compounds, and developing standardized dosage guidelines would substantially enhance the safety profile of their clinical applications. Furthermore, the majority of current investigations on AS remain at the preclinical stage. Substantial physiological and metabolic disparities—including differences in lipid metabolism, immune regulation, and drug processing—exist between animal models and humans. Moreover, prevailing animal studies often employ *in vitro* administration protocols, which fail to adequately recapitulate the complex pharmacokinetic behavior in living systems. Cellular models, meanwhile, are limited by their tendency to simulate only isolated aspects of AS pathogenesis, thus lacking organism-level physiological relevance. Therefore, well-designed controlled clinical trials in human populations—provided that safety criteria are strictly met—represent a more reliable approach for evaluating the efficacy of plant-derived compounds. Alternatively, direct mechanistic validation using human-derived tissue samples may help establish a clearer causal relationship among phytochemical intervention, PI3K/AKT signaling pathway modulation, and AS pathological progression. Finally, application of the PAINS filter revealed that a considerable number of plant-derived compounds summarized in this review are classified as PAINS, for instance, berberine, kaempferol, and rutin. These compounds are prone to yield non-specific positive results in experimental assays, substantially undermining the reliability of their purported biological effects. Therefore, constructing specialized protein databases to analyze PAINS-related protein–ligand structures, along with elucidating their chemical characteristics and variable binding modes, will facilitate the discrimination of PAINS compounds that genuinely exert therapeutic effects against atherosclerosis (Bolz et al., 2021).

### 6.3 Future research directions

We propose the following recommendations for future research: 1. low bioavailability significantly limits the therapeutic efficacy of plant-derived compounds upon their conversion into pharmaceutical agents. This limitation may be addressed by developing novel drug delivery systems, such as nanoparticles formulated using extracellular vesicles, or targeted liposomal preparations incorporating plant-derived compounds. 2. To precisely elucidate the toxic and side-effect profiles of plant-derived compounds and establish standardized dosage regimens, it is imperative to conduct comprehensive pharmacological analyses. This approach should integrate advanced techniques, such as network pharmacology and molecular docking, to aid in the determination of underlying mechanisms, complemented by systematic long-term toxicity assessments utilizing diverse animal models and cellular systems to ensure a reliable safety evaluation. 3. Current research on plant-derived compounds for the treatment of AS remains predominantly at the basic experimental stage. To enhance clinical translatability, future studies may employ humanized animal models that recapitulate the pathophysiological milieu of AS. Alternatively, directly conducting randomized controlled trials or blinded clinical studies would help identify the precise targets of these compounds in humans. 4. The active constituents of plant-derived compounds are highly complex; thus, future investigations should adopt integrated multi-omics strategies—such as genomics, transcriptomics, metabolomics, and proteomics—to elucidate the biosynthetic pathways underpinning their efficacy. For instance, a combined metabolomics and proteomics approach has been applied to reveal the interactive mechanism between tea polyphenols and gut microbiota (Kim et al., 2025). 5. There is a prevalent lack of rigor in mechanistic validation and insufficient awareness of PAINS. It is recommended that future studies subject plant-derived compounds reported to modulate the PI3K/AKT pathway in AS therapy to systematic PAINS filtering. Furthermore, more stringent pharmacological methods should be employed—such as using non-ionic detergents to disrupt colloidal aggregates and prevent nonspecific protein co-precipitation or designing rescue experiments via downstream effectors of the PI3K/AKT signaling cascade—to verify target specificity and enhance the reliability of conclusions.

### 6.4 Clinical translation prospects

A diverse array of plant-derived compounds—including terpenoids, flavonoids, alkaloids, polysaccharides, stilbenes, and phenylpropanoids—can be isolated from botanical sources. In contrast to conventional single-target chemical drugs, natural compounds exhibit a multi-target and multi-pathway synergistic therapeutic profile, enabling modulation of AS across its various pathological stages. Among the 29 natural compounds systematically reviewed in this study, several demonstrate considerable clinical potential. For instance, artemisinin is distinguished by its multi-target engagement and favorable safety profile, while berberine shows marked efficacy in regulating lipid metabolism and attenuating inflammatory responses. These attributes endow them with high translational promise, positioning them as candidates for developing more effective and safer therapeutic options for patients.

## 7 Conclusion

In summary, although challenges remain regarding the administration, bioavailability, and toxicological profiling of plant-derived compounds in the treatment of AS, substantial evidence supports their considerable therapeutic potential through modulation of the PI3K/AKT signaling pathway, thereby influencing inflammation, metabolism, and cellular proliferation. This review is anticipated to furnish novel perspectives and a theoretical foundation for the management of AS and other cardiovascular diseases.
